# Allosteric Modulation of PS1/γ-Secretase Conformation Correlates with Amyloid β_42/40_ Ratio

**DOI:** 10.1371/journal.pone.0007893

**Published:** 2009-11-18

**Authors:** Kengo Uemura, Christina M. Lill, Xuejing Li, Jessica A. Peters, Alexander Ivanov, Zhanyun Fan, Bart DeStrooper, Brian J. Bacskai, Bradley T. Hyman, Oksana Berezovska

**Affiliations:** 1 Alzheimer Research Unit, MassGeneral Institute for Neurodegenerative Diseases, Massachusetts General Hospital, Charlestown, Massachusetts, United States of America; 2 Laboratory of Neuronal Cell Biology and Gene Transfer, Katholieke Universiteit Leuven, Leuven, Belgium; Tokyo Medical and Dental University, Japan

## Abstract

**Background:**

Presenilin 1(PS1) is the catalytic subunit of γ-secretase, the enzyme responsible for the Aβ C-terminal cleavage site, which results in the production of Aβ peptides of various lengths. Production of longer forms of the Aβ peptide occur in patients with autosomal dominant Alzheimer disease (AD) due to mutations in presenilin. Many modulators of γ-secretase function have been described. We hypothesize that these modulators act by a common mechanism by allosterically modifying the structure of presenilin.

**Methodology/Principal Findings:**

To test this hypothesis we generated a genetically encoded GFP-PS1-RFP (G-PS1-R) FRET probe that allows monitoring of the conformation of the PS1 molecule in its native environment in live cells. We show that G-PS1-R can be incorporated into the γ-secretase complex, reconstituting its activity in PS1/2 deficient cells. Using Förster resonance energy transfer (FRET)-based approaches we show that various pharmacological and genetic manipulations that target either γ-secretase components (PS1, Pen2, Aph1) or γ-secretase substrate (amyloid precursor protein, APP) and are known to change Aβ_42_ production are associated with a consistent conformational change in PS1.

**Conclusions/Significance:**

These results strongly support the hypothesis that allosteric changes in PS1 conformation underlie changes in the Aβ_42/40_ ratio. Direct measurement of physiological and pathological changes in the conformation of PS1/γ-secretase may provide insight into molecular mechanism of Aβ_42_ generation, which could be exploited therapeutically.

## Introduction

γ-Secretase is an enzymatic complex composed of at least four proteins: presenilin 1 or presenilin 2 (PS1 or PS2), Nicastrin, Pen2 and Aph1 [1]–[5], with presenilin representing the catalytic core [6]–[9]. Change in the precision of the APP substrate cleavage by γ-secretase result in change in the Aβ_42/40_ ratio. Aβ_42_ represents a highly fibrillogenic species that is more prone to form neurotoxic oligomers leading to synaptic dysfunction and neuronal death [10], [11]. An increase in the Aβ_42/40_ ratio has been implicated in Alzheimer's disease (AD) pathogenesis. However, it remains unclear what cellular and molecular factors are responsible for the shift in APP γ-cleavage site towards toxic Aβ_42_ species.

A number of genetic and pharmacological manipulations targeting either γ-secretase complex or its APP substrate have been demonstrated to alter Aβ_42/40_ ratio. For example, familial AD-linked mutations in PS1, elongation of Pen2 N-terminus (NT), or expression of Aph1B isoform, increase the Aβ_42/40_ ratio [12]–[14]. Targeting the APP substrate directly by introducing mutations near the APP γ-secretase cleavage site [15], [16] modifies Aβ_42/40_ ratio. In addition, a subset of Aβ_42_-lowering non-steriodal anti-inflammatory drugs [17], [18] act as γ-secretase modulators (GSMs) indirectly via binding to the APP substrate [19]. These APP-targeting manipulations may alter APP conformation, its positioning within the membrane, APP substrate presentation to γ-secretase, and/or stabilize PS1 in a certain conformation, and thus affect the site of APP cleavage by γ-secretase.

In the present study we test the hypothesis that Aβ_42/40_ ratio correlates with allosteric PS1/γ-secretase conformation. We have previously developed an assay to monitor PS1 conformation in fixed and permeabilized cells by labeling different domains of PS1 with fluorophore-tagged antibodies. Based on our findings [20], [21] we hypothesize that the mature PS1 molecule may exist in equilibrium of two conformational states: “open” and “closed”, corresponding to a predominant cleavage of APP at Aβ_40_ and Aβ_42_ sites, respectively. To test the hypothesis that multiple GSMs act by allosterically influencing PS1 conformation, and to directly monitor PS1 conformation in live cells, we developed a new assay that monitors proximity between the PS1 NT and the major cytosolic loop between PS1 transmembrane domains 6 and 7 (TM6-7 loop). For this, a GFP-PS1-RFP (G-PS1-R) construct was generated by tagging PS1 NT with green fluorescent protein (GFP) and TM6-7 loop with red fluorescent protein (mRFP). We demonstrate that various Aβ_42_ raising manipulations, including direct manipulations of the γ-secretase components (Pen2, Aph1 and PS1) or pharmacological targeting of APP (fenofibrate) lead to a common conformational change of PS1: closer proximity of the PS1 NT and TM6-7 loop. Conversely, treatment with the Aβ_42_–lowering agent (ibuprofen) moves PS1 NT and loop farther apart.

Thus, we found that a broad range of Aβ_42_-altering manipulations, regardless of their precise molecular mechanism or their target, lead to a consistent change in PS1 conformation. This suggests that PS1 conformation could be a reliable predictor of Aβ production, and presents an allosteric modulation of the “pathogenic” PS1 conformation as an attractive therapeutic strategy.

## Results

### GFP-PS1-RFP Probe Reconstitutes γ-Secretase Activity

**Figure 1:**
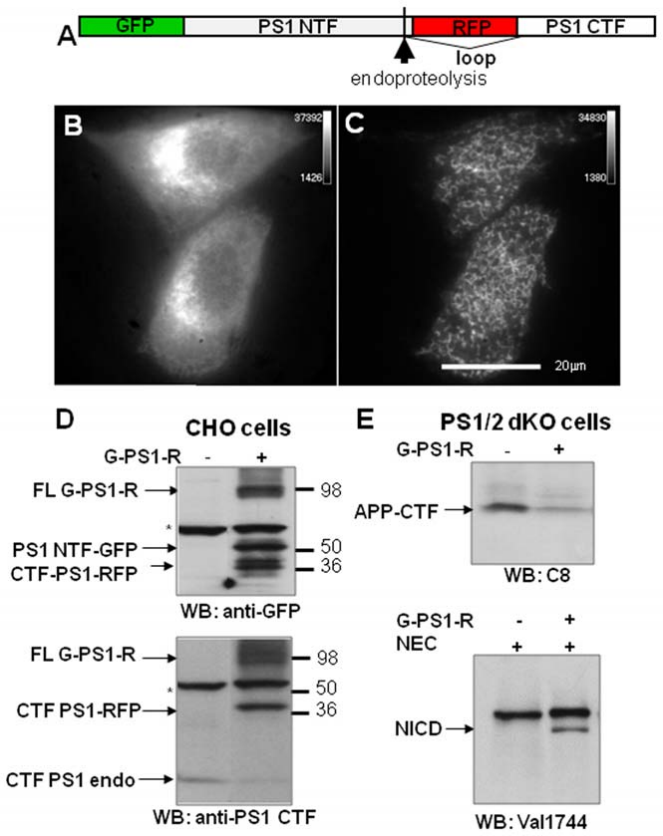
Expression, processing, and activity of the G-PS1-R protein. (**A**)- Schematic representation of the G-PS1-R construct. The GFP is fused to the N-terminus of PS1, and serves as a donor fluorophore in the FRET assays, whereas RFP (an acceptor fluorophore) is introduced by PCR into the TM6-7 loop region of PS1 between amino acids 351 and 352. (**B–C**) The expression of the G-PS1-R in CHO cells. (**B**) -Epi-fluorescent signal shows strongest fluorescence intensity in the perinuclear region of the cell by confocal microscopy. (**C**) - Cell surface expression of the G-PS1-R probe shown by TIRFM. A speckled pattern of the TIRF signal indicates that G-PS1-R protein reaches the cell surface. (**D**) CHO cells were transfected with G-PS1-R construct and cell lysates were subjected to the western blot analysis. (**D, Top**) G-PS1-R undergoes endoproteolysis to yield PS1 NTF and CTF. A ∼100 kDa full-length G-PS1-R, ∼50 kDa PS1 NTF-GFP and ∼40 kDa CTF PS1-RFP were observed by blotting with the anti-GFP antibody. Asterisk: non-specific bands. (**D, Bottom**) Blotting by anti-PS1 CTF is shown. Full-length G-PS1-R as well as ∼40 kDa PS1-RFP CTF were detected. Note, that endogenous PS1 CTF is reduced in the G-PS1-R expressing CHO cells (replacement phenomena). (**E**) G-PS1-R reconstitutes γ-secretase activity in PS1/2 dKO MEF cells. (**E, Top**) APP CTF is reduced in PS1/2 dKO MEF cells expressing G-PS1-R; probed with anti-APP CTF antibody. (**E, Bottom**) PS1/2 dKO cells were transfected with NEC with or without G-PS1-R construct, lysed, and analyzed by western blotting using NICD-specific (Val1744) antibody. Note that NICD can only be observed in cells expressing G-PS1-R.

GFP-PS1-RFP (G-PS1-R) construct was generated to monitor PS1 conformation (PS1 NT-loop proximity) in live cells. For this, green fluorescent protein (GFP) was fused to the PS1 N-terminus and red fluorescent protein (mRFP) was inserted into the TM6-7 loop domain (**Figure 1A**). The resulting G-PS1-R fusion protein represents an ideal molecular probe for FRET-based analysis with a donor and an acceptor fluorophore in a 1∶1 ratio. When expressed in cells, the G-PS1-R probe shows perinuclear distribution of GFP and RFP, a pattern similar to that of endogenous wt PS1 (**Supplementary Figure S1,** and **Figure 1B**). To examine whether the G-PS1-R probe can traffic though the secretory pathway to the plasma membrane, we assessed the expression pattern of the G-PS1-R protein in cells by using total internal reflection fluorescence microscopy (TIRFM), which detects fluorescent signal within 100 nm of the cell surface. A dotty pattern of the TIRFM signal was detected at the cell surface in G-PS1-R expressing cells, indicating that a few percent of the G-PS1-R molecules reach the cell surface (**Figure 1C**).

Next, we asked whether the G-PS1-R probe could be incorporated into the γ-secretase complex to exert its enzymatic function. To test this, we monitored the ability of G-PS1-R to undergo endoproteolysis, and to cleave APP and Notch γ-secretase substrates. First, CHO cells were transiently transfected with G-PS1-R or infected with lenti-G-PS1-R, lysed, and analyzed by Western blot. As shown in **Figure 1D**, G-PS1-R undergoes endoproteolysis to generate ∼60 kDa PS1 NTF-GFP and ∼40 kDa CTF-RFP. Noticeably, the endogenous PS1 was reduced in cells transfected with G-PS1-R, suggesting that other components of the γ-secretase are forming a complex with exogenous G-PS1-R, destabilizing endogenous wild-type (wt) PS1 (the ‘replacement phenomena’) (**Figure 1D, bottom panel**).

To determine if G-PS1-R is able to reconstitute γ-secretase activity, PS1/2 dKO MEF cells were transfected with G-PS1-R, and APP processing was assessed by monitoring APP C-terminal fragment (CTF) levels in cell lysate. Using an APP C-terminal specific antibody, we observed the reduction in the level of APP CTFs in PS1/2 dKO cells transfected with G-PS1-R compared to that in the “no PS” lane (**Figure 1 Etop panel** ). This indicates that APP CTFs, immediate substrates of the γ-secretase, are cleaved by G-PS1-R containing γ-secretase complex. Furthermore, when PS1/2 dKO MEF cells were transfected with another immediate substrate of γ-secretase, the constitutively active NEC construct, the cleavage product Notch intracellular domain (NICD) was detected only in G-PS1-R co-transfected cells (**Figure 1E bottom panel** ). Thus, we conclude that G-PS1-R can assemble into the γ-secretase complex and is able to reconstitute γ-secretase activity in PS1/2 dKO cells.

### Detection of Inter- versus Intra-Molecular FRET Using G-PS1-R Probe

To test whether GFP on the N-terminus and RFP in the loop domain of PS1 are close enough to support FRET, CHO cells were transfected with GFP-PS1 (negative control) or G-PS1-R constructs, and analyzed by Fluorescence Lifetime Imaging Microscopy, FLIM. First, the baseline lifetime of the GFP donor was measured in GFP-PS1 transfected cells in the absence of RFP acceptor (**Table 1**). As expected, GFP lifetime significantly shortens in the presence of the RFP acceptor in G-PS1-R transfected cells, compared to that in the G-PS1 expressing cells. This indicates FRET, i.e. close proximity between GFP-PS1 NT and RFP-PS1 loop. Since PS1 dimers have been previously reported, [22]-[24] we tested how much of the FRET signal is contributed by inter-molecular (PS1 dimers) versus intra-molecular (single PS1 molecule) interaction. For this, dilution of the G-PS1-R probe with untagged PS1 DNA was prepared for the transfection. We reasoned that dilution of the G-PS1-R probe by the wt PS1 would reduce inter-molecular FRET via competitive inhibition of the G-PS1-R/G-PS1-R dimer formation, whereas G-PS1-R intra-molecular FRET would not be affected. GFP-PS1 NT to RFP-PS1 loop FRET efficiency (GFP lifetime shortening) was monitored by FLIM in cells transfected with G-PS1-R only (presumed mixture of intra- and inter-molecular FRET), co-transfected with G-PS1-R and untagged wild type PS1 in 1∶3 ratio (mostly intra-molecular FRET), and in cells co-transfected with GFP-PS1 and PS1-RFP constructs (inter-molecular FRET only). We found that the GFP lifetime did not significantly change when G-PS1-R was diluted by unlabeled PS1, indicating that the vast majority of the detected FRET signal was due to intra-molecular FRET (**Table 1**). In addition, GFP-PS1 lifetime in GFP-PS1 and PS1-RFP co-transfected cells (inter-molecular FRET) was significantly longer, comparing to that in the G-PS1-R transfected cells. This further supports the notion that the FRET signal detected with the G-PS1-R probe originates mostly from intra-molecular FRET.

**Table 1:** Detection of inter- vs. intra-molecular FRET using G-PS1-R probe.

Condition	Donor	Acceptor	Donor lifetime (psec)
**G-PS1**	GFP-NT	none	2097±78 (n = 11)
**G-PS1-R**	GFP-NT	RFP-loop	1783±197 (n = 14)*
**G-PS1-R∶wtPS1 (1∶3)**	GFP-NT	RFP-loop	1752±117 (n = 14)*
**G-PS1+PS1-R (1∶1)**	GFP-NT	RFP-loop	1983±62 (n = 13)#

* : p<0.001 vs G-PS1,

#: p<0.05 vs G-PS1.

### Manipulation of the γ-Secretase Components Leads to Altered Aβ_42/40_ Ratio and Correlates with PS1 Conformational Change

Based on our previous findings in fixed cells, we have hypothesized that mature PS1 molecules may exist in at least two conformational states: “open” and “closed” [20], [21]. According to this model, familial Alzheimer's disease (FAD) PS1 mutations shift the equilibrium to favor a “close” NT-CT conformation (with NT-CT closer together) and stabilize an alignment of the PS1/γ-secretase active site with the APP substrate to favor predominant cleavage of APP at the Aβ_42_ position. Now we test whether the G-PS1-R probe can monitor changes in PS1 conformation in live cells.

First, to determine if the G-PS1-R probe adopts a different conformation in the presence of FAD-linked mutation, either wild-type G-PS1-R probe or G-PS1-R probe incorporating the L166P PS1 mutation (L166P G-PS1-R) was transfected into CHO cells and the NT-loop proximity was analyzed by FLIM. We found that the GFP lifetime in the L166P G-PS1-R expressing cells was noticeably shorter (**Table 2**), compared to that in the wt G-PS1-R transfected cells, indicating that L166P G-PS1-R adopts a “close” NT-loop conformation.

**Table 2:** The effect of L166P mutant on G-PS1-R conformation.

Condition	Donor	Acceptor	Donor lifetime (psec)
**G-PS1**	GFP-NT	none	2074±27 (n = 7)
**G-PS1-R**	GFP-NT	RFP-loop	1742±244 (n = 15)
**L166P G-PS1-R**	GFP-NT	RFP-loop	1488±145 (n = 15)*

* : p<0.001 vs G-PS1-R.

Isoo et al. [13] reported recently that introducing a structural change to the N-terminus of one of the γ-secretase complex components, Pen2, leads to an increase in the Aβ_42_ production. Since we have previously observed that a change in the Aβ_42/40_ ratio due to familial AD mutations correlates with a change in the PS1 NT-CT proximity we hypothesized that an elongation of the Pen2 NT may have an effect on PS1 conformation similar to that of FAD PS1 mutations.

To test this, CHO cells were co-transfected with G-PS1-R and either wt Pen2 or Pen2 with elongated NT due to a flag tag (flag-Pen2). We found that in the conditioned media of the flag-Pen2 transfected cells the Aβ_42/40_ ratio was increased by ∼1.6 fold, compared to that in the Pen2 transfected cells, confirming the data by Isoo et al. [13]. Correspondingly, when we monitored G-PS1-R conformation in living cells by the FLIM assay we found that cells co-transfected with flag-Pen2 displayed closer PS1 NT-loop proximity compared to that in the wt Pen2 transfected cells, as reflected by the shortening of the average donor fluorophore lifetime in flag-Pen2 expressing cells (**Table 3**). We used an alternative approach to confirm these findings; our previously validated PS1 NT-CT conformation FLIM assay in fixed and immunostained cells. The cells were double-immunostained with the previously reported PS1 NT-CT pair of antibodies [21] as well as with the PS1 NT-loop pair of antibodies, a new assay that matches the G-PS1-R NT-loop conformation assay. The primary antibodies were fluorescently labeled with Alexa488 (A488) as a donor and Cy3 as an acceptor fluorophores. We found a statistically significant shortening of the donor lifetime per cell in the flag-Pen2 transfected cells in both PS1 NT-CT and PS1 NT-loop assays (**Table 3**). This indicates that elongation of the Pen-2 N-terminus correlates with the change in PS1 conformation, which in turn leads to an increase in Aβ_42_ generation.

**Table 3:** Manipulation of Pen-2 lead to altered Aβ_42/40_ ratio as well as PS1 conformation.

Condition	Donor	Acceptor	Donor lifetime (psec)
**wt/flag-Pen2**	GFP-NT	none	2017±22 (n = 12)
**wt Pen2**	GFP-NT	RFP-loop	1719±135 (n = 19)
**flag-Pen2**	GFP-NT	RFP-loop	1430±194 (n = 22)*
**wt/flag-Pen2**	Alexa488-NT	none	2223±14 (n = 6)
**wt Pen2**	Alexa488-NT	Cy3-loop	1852±59 (n = 10)
**flag-Pen2**	Alexa488-NT	Cy3-loop	1799±89 (n = 10)**
**wt Pen2**	Alexa488-NT	Cy3-CT	1802±61 (n = 10)
**flag-Pen2**	Alexa488-NT	Cy3-CT	1699±109 (n = 10)***

* : p<0.05 vs wt Pen2,

** : p<0.05 vs wt Pen2,

*** : p<0.05 vs wt Pen2.

We have recently reported that different isoforms of another γ-secretase component, Aph1 (Aph1A vs. Aph1B), support differential γ-secretase activity, with Aph1B containing γ-secretase complexes yielding a higher Aβ_42/40_ ratio [14]. Using the antibody-based FLIM assay we showed that PS1 has shorter distance between its NT and CT in cells containing Aph1B alone, compared to that in Aph1A expressing cells [14]. Thus, we asked whether conformational changes in the PS1 molecule induced by different Aph1 isoforms could be detected in live cells by the G-PS1-R probe. MEF cells expressing either all Aph1 isoforms (wt Aph1), or exclusively Aph1A or Aph1B containing γ-secretase complexes were transfected with G-PS1-R, and NT-loop distance was evaluated by the FLIM analysis. We found that the lifetime of the GFP-donor was significantly shorter in the Aph1B containing cells, compared to that in fibroblasts expressing Aph1A or wild-type Aph1 (**Table 4**). These results further support the notion that PS1 adopts a “close” conformation in live cells expressing Aph1B-containing γ-secretase complexes, and such PS1 conformation may be responsible for a shift towards the Aβ_42_ cleavage site.

**Table 4:** Manipulation of Aph1 lead to altered PS1 conformation.

Condition	GFP donor	RFP acceptor	Donor lifetime (psec)
**wt fibroblast**	GFP-NT	none	2110±35 (n = 26)
**wt fibroblast**	GFP-NT	RFP-loop	1867±80 (n = 22)
**Aph1A**	GFP-NT	RFP-loop	1874±58 (n = 22)
**Aph1B**	GFP-NT	RFP-loop	1766±84 (n = 22)*

* : P<0.001 vs wt, Aph1A.

### Pharmacological Manipulations of the Aβ_42_ Production Are Associated with a Change in G-PS1-R Conformation

Several compounds, referred to as γ-secretase modulators (GSMs), have been shown to selectively affect production of Aβ species of different lengths by affecting the APP γ-secretase cleavage site. Weggen et al. [17] reported that the group of NSAIDs shifted the γ-secretase cleavage site on APP from the Aβ_42_ position to Aβ_38_, selectively reducing Aβ_42_ generation. Another set of compounds, such as fenofibrate, mimicked the effect of FAD mutations by increasing Aβ_42_ production [13], [25]. Now we explore whether we can detect G-PS1-R conformational change in live cells due to GSM treatment. For this, CHO cells transfected with G-PS1-R probe were treated with either 100 µM fenofibrate or DMSO. The conditioned media was collected for Aβ specific ELISA and the cells were used for the analysis of PS1 NT-loop proximity by FLIM. The Aβ ELISA revealed that in cells treated with fenofibrate the level of secreted Aβ_40_ was reduced by ∼42% and the level of Aβ_42_ was increased by ∼36%. FLIM analysis showed that fenofibrate treatment leads to a significant shortening of the GFP lifetime in cells expressing G-PS1-R construct, compared to that in DMSO treated cells (**Table 5**), indicating a decrease in the GFP-PS1-NT and RFP-PS1-loop proximity.

**Table 5:** Pharmacological modulation of Aβ_42_ by fenofibrate changes conformation of G-PS1-R.

Condition	Donor	Acceptor	Donor lifetime (psec)
**DMSO/Fenofibrate**	GFP-NT	None	2013±8 (n = 6)
**DMSO**	GFP-NT	RFP-loop	1772±63 (n = 11)
**Fenofibrate**	GFP-NT	RFP-loop	1666±26* (n = 10)
**DMSO/Fenofibrate**	Alexa488-NT	none	2083±36 (n = 7)
**DMSO**	Alexa488-NT	Cy3-CT	1823±25 (n = 15)
**Fenofibrate**	Alexa488-NT	Cy3-CT	1698±58** (n = 16)

* : p<0.05 vs DMSO,

** : p<0.02 vs DMSO.

The representative experiment is shown; total four independent experiments were performed.

Similarly, our previously validated endogenous PS1 NT-CT conformation FLIM assay in fixed and immunostained cells showed statistically significant shortening of the donor (Alexa 488) lifetime in cells treated with fenofibrate, compared to that in the DMSO treated cells (**Table 5**). These data suggest that fenofibrate alters the conformation of the PS1 molecule to favor a “close” PS1 NT-loop and PS1 NT-CT proximity, which correlates with a shift in the APP γ-cleavage site.

### Detection of the PS1 Conformation in Live Cells by Spectral FRET

**Figure 2:**
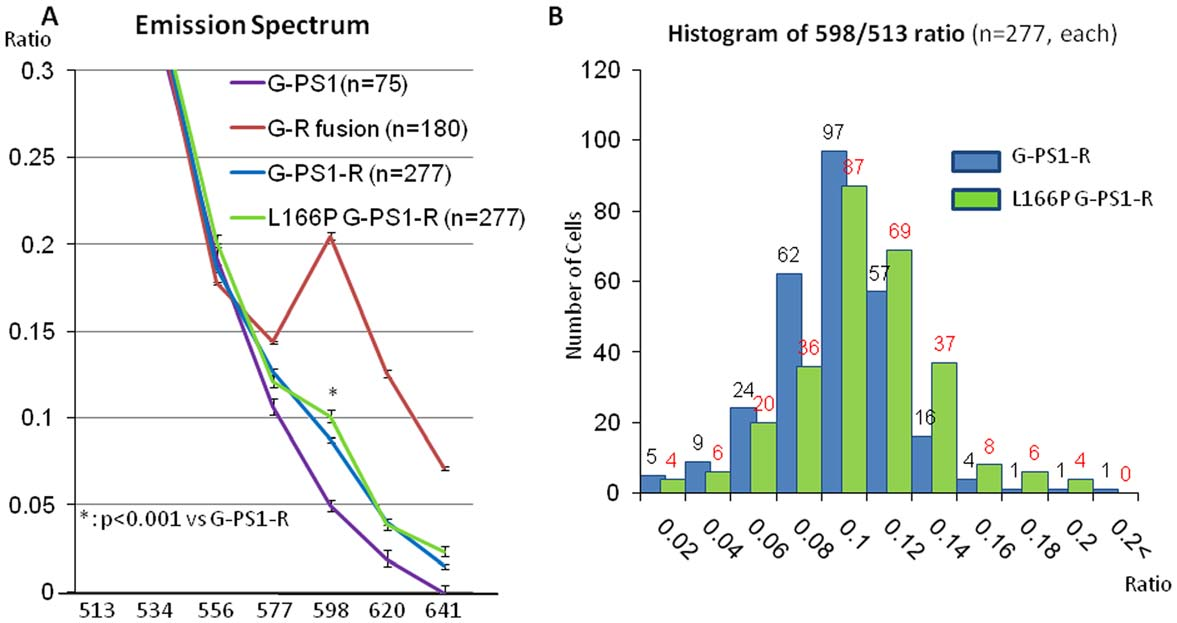
Detection of the PS1 conformation in living cells by spectral FRET. (**A**) HEK293T cells were transfected with designated constructs and activated by the 488 nm laser. Vertical axis shows the ratio of intensities within the 513 nm to 641 nm spectral range normalized by the intensity at 513 nm. The GFP-RFP fusion construct (red) shows large ‘hump’ centered on 598 nm, compared to G-PS1 (purple) fluorescence intensity due to FRET, i.e., energy transfer from GFP to RFP. L166P G-PS1-R (green) shows smaller but significant (p<0.001) increase in the 598/513 ratio, compared to that in the wild-type G-PS1-R expressing cells (blue). (**B**) HEK293Tcells were transfected with either G-PS1-R or L166P mutant G-PS1-R construct. Equal numbers of cells (n = 277) were randomly chosen from each condition and cell number was plotted against the 598/513 ratio. Note that the histogram is shifted to the right for L166P mutants, indicating an increase in the number of cells that exhibit higher 598/513 ratio, i.e., closer PS1 NT-loop proximity (p<0.001).

While FLIM approach provides high spatial resolution, each FLIM image takes minutes to acquire. The high temporal resolution of Spectral FRET analysis allows detection of more rapid changes in the FRET efficiency in live cells transfected with fluorescent proteins and enables monitoring these changes on a high throughput basis. First, we established optimal settings for the spectral imaging of the G-PS1-R transfected cells, and selected spectral windows in which changes in the donor and acceptor fluorescence intensity due to FRET are the most evident (**Figure 2A**). A construct encoded by RFP fused to GFP in a 1∶1 ratio via a short linker (R-G fusion) was used as a positive control of the energy transfer, whereas G-PS1 (no acceptor) was used as a negative control. When cells transfected with the R-G fusion construct were activated by a 488 nm laser, the emission spectrum changed from that of the G-PS1, yielding a large spike at the 598 nm as a result of the energy transfer from the GFP donor to the RFP acceptor. Thus, we determined the ratio of fluorescence intensity at the 598 nm to that at the 513 nm (emission peak of the GFP) as a readout for the FRET efficiency: higher 598/513 ratio indicates higher FRET efficiency, and closer proximity between the fluorophores.

When cells transfected with the G-PS1-R probe were activated by the 488 nm laser, we observed a shift in the emission spectra, indicating the presence of FRET from GFP to RFP, and resulting in a significantly higher 598/513 ratio, compared to that of the G-PS1 control. Next, we confirmed that the spectral FRET assay can detect subtle conformational differences between the wild type and L166P mutant G-PS1-R molecules. As shown in **Figure 2A**, the emission spectrum curve of the L166P G-PS1-R showed a small ‘hump’ at 598 nm, indicating a higher FRET efficiency compared to that of the wild type G-PS1-R. We observed a small but statistically significant increase in the average 598/513 ratio distribution in cells expressing the FAD mutant PS1 (**Figure 2B**). This result indicates that in the presence of the L166P mutation, GFP at the PS1 NT is closer to or more directly aligned with the RFP in the TM6-7 loop, giving rise to more efficient energy transfer from G to R, compared to that in the wt PS1 molecules.

To visualize structural differences between the G-PS1-R molecules in different subcellular compartments of the same cell, pseudo-colored ratiometric images of the 598/513 intensity were generated by Image J program after background subtraction. Several regions of interest (ROI) were randomly selected within the cell expressing G-PS1-R (**Supplementary Figure S2A**) and the 598/513 ratio was calculated in each ROI. As expected, ROIs located at the cell periphery yield higher 598/513 ratio (more green-to-red pixels in the pseudo-colored Spectral FRET image), compared to the ROI adjacent to the nucleus (predominantly blue pixels, **Supplementary Figure S2B**). This distribution is very similar to that in HEK cells transfected with G-PS1-R and analyzed by FLIM (**Supplementary Figure S2C**). The pseudo-colored FLIM image shows shorter lifetime of the donor GFP at the periphery of the cells (red pixels), indicating ‘closer’ NT-loop conformation of the G-PS1-R probe at/near the cell surface. Thus, both FLIM and spectral FRET imaging in live cells revealed that G-PS1-R molecules adopt more “close” NT-loop conformation as they move towards the cell surface, a pattern which is identical to that of previously observed endogenous PS1 [21].

**Figure 3:**
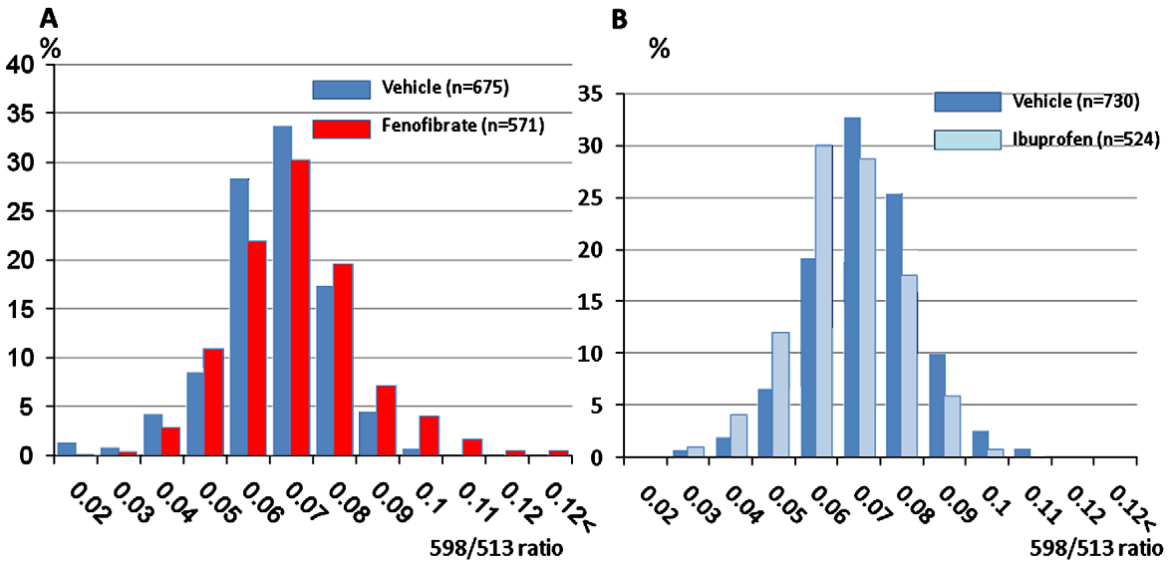
Effect of GSMs on G-PS1-R conformation as detected by spectral FRET. HEK293 cells infected with G-PS1-R-lentiviral construct were treated with either (A) vehicle (DMSO) or 100 µM fenofibrate, or (B) vehicle (EtOH) or 300 µM ibuprofen for 24 hours. After the treatment, cells were activated by the 488 nm laser and analyzed by spectral FRET. The percentage of cells exhibiting certain 598/513 ratio (Y axis) was plotted against the 598/513 ratio (X axis). (**A**) Note that the histogram in the fenofibrate treated cells is shifted to the right of that in DMSO treated cells. This indicates a higher percentage of cells with elevated 598/513 ratio after the fenofibrate treatment (p<0.001 vs. DMSO; DMSO: n = 675 cells, fenofibrate: n = 571cells). (**B**) Note that higher percentage of cells shows lower 598/513 ratio after the ibuprofen treatment (p<0.001 vs. ethanol; ethanol: n = 730 cells, ibuprofen: n = 524cells).

Finally, we asked if the effect of GSMs treatment on PS1 conformation could be detected by spectral FRET in live cells. To test this, HEK293 cells were infected with G-PS1-R-lenti viral constructs. 24 hrs after the infection, cells were treated with either 100 µM fenofibrate (closes PS1 conformation) or 400 µM ibuprofen (opens PS1 conformation [20]) for 24 hours followed by the 598/513 ratio analysis. Control cells were treated with vehicle (DMSO and ethanol, respectively). As expected, fenofibrate treatment resulted in a spectral shift similar to that observed for the L166P G-PS1-R construct. We detected a significant increase in the 598/513 ratio in the presence of fenofibrate (**Figure 3A**), which indicates that fenofibrate treatment induces the “closed” conformation of PS1.Conversely, ibuprofen treatment leads to a reduced 598/513 ratio (**Figure 3B**), inducing an “open” PS1 conformation. Thus, G-PS1-R conformation changes in response to agents that selectively modulate Aβ_42_ production in live cells.

## Discussion

An aggregation and deposition of amyloid β (Aβ) containing plaques is a common feature of normal aging and the AD brain. A shift towards an increased proportion of the longer, highly fibrillogenic and neurotoxic Aβ species (i.e., Aβ_42_) in the brain has been associated with the neurodegeneration leading to AD. Blocking Aβ production can be a therapeutic target. However, direct inhibition of γ-secretase activity may cause undesirable side effects due to disruption of the normal physiological function of numerous γ-secretase substrates, including APP itself [26] and Notch [27]. Instead, modulators of the site at which γ−secretase cleaves APP have been developed by screening libraries of compounds, or noted by genetic manipulations [13], [17], [25]. However, the precise molecular and cellular mechanism underlying the resultant changes in the Aβ_42/40_ ratio is unknown. We propose a hypothesis of “structure-function correlation” implying that structural changes in the PS1/γ-secretase are linked to the precision of γ-cleavage of APP to yield Aβ species of different lengths. Our data suggest that changes in the structure of multiple members of the γ-secretase complex, or even APP itself, can allosterically impact PS1 conformation in a way that leads to a change in γ-secretase/APP interactions and alters the exact cleavage site of APP.

Very little is currently known about the structure of the PS1/γ-secretase complex due to its complexity and the difficulty of crystallizing multi-pass membrane proteins. Several studies that use serial deletions and co-immunoprecipitation [28], substituted cysteine accessibility method [13], [29], [30] or electron microscopy-based analyses [31] reported that PS1/γ-secretase may form a ring-like pore within the hydrophobic environment of a membrane. However, all these studies required lysing of the cells and/or were performed using isolated γ-complexes. To test the hypothesis of PS1/γ-secretase structure-function correlation in living cells and to accurately report molecular changes in PS1 in its native environment, a fluorescent probe is required that adequately responds to various experimental manipulations associated with alterations in the Aβ_42/40_ ratio. In this report, we present and fully characterize a novel probe, GFP-PS1-RFP fusion protein, which exploits NT-loop proximity as a readout of PS1 conformation in live cells. We employ a molecular imaging methodology, such as FRET-based microscopy, to show that this probe allows monitoring, and accurately reflects consistent structural changes of the PS1 molecule in live cells, and that these changes closely correlate with changes in the Aβ_42/40_ ratio.

We verified that G-PS1-R protein can be cleaved into NTF and CTF, can traffic to the plasma membrane, which is critical for the metabolism of some substrates, and can be incorporated into the γ-secretase complex to support enzymatic activity in cells lacking both presenilin 1 and presenilin 2. Moreover, using both FLIM and spectral FRET approaches we determined that G-PS1-R protein adopts “close” or “open” PS1 NT-loop proximity in response to various Aβ_42_-raising or lowering manipulations, respectively. These manipulations include introducing familial Alzheimer's disease L166P PS1 mutation, structural change in the Pen2 N-terminus, expressing Aph1B or Aph1A isoform, or pharmacological treatment (fenofibrate, ibuprofen). We show that data obtained using G-PS1-R probe are in agreement with our previous findings using FLIM analysis of fixed, permeabilized, and immunostained cells, that utilized either NT-CT or NT-loop proximity to monitor PS1 conformation [14], [20], [21]. Since antibody-based FLIM analysis is based on immunolabeling of endogenous and/or untagged PS1 molecules, these results confirm that the G-PS1-R probe is a reliable indicator of PS1 conformation in living cells.

The finding that the G-PS1-R probe acquires “open” conformation in response to ibuprofen treatment and “close” NT-loop proximity in response to fenofibrate is very interesting since it has recently been shown that these agents target APP substrate [19], and not γ-secretase complex itself as previously believed [32], [33]. This indicates that modifications in the substrate could allosterically affect conformation of the enzyme. In line with this finding, we have previously showed that APP mutations which reduce Aβ_42_ lead to an “open” [15], whereas mutations that increase Aβ_42_ induce “close” [16] conformation of the PS1 molecule. Thus, altered presentation of the APP substrate to γ-secretase, driven by either APP mutations or by binding of the pharmacological agents to APP could, in turn, allosterically affect PS1 conformation.

Collectively, our data demonstrate that regardless of the nature (genetic or pharmacological GSMs) or precise target (i.e. PS1, Pen-2, Aph-1 or APP substrate), the final determinant of Aβ_40_ or Aβ_42_ generation relies on the molecular conformation of PS1. These findings strongly support the hypothesis that Aβ_42/40_ ratio is tightly linked to the conformation of the PS1 molecule, thus making allosteric modulation of “pathogenic” conformation of PS1 an attractive target of therapeutic intervention in Alzheimer's disease.

In addition, we present a ratiometric spectral FRET assay that can be used for screening drugs modulating Aβ_42/40_ ratio on a high-throughput screen platform and/or in live mouse brain *in vivo*. An additional advantage of using G-PS1-R probe in this assay is that it allows monitoring PS1 conformational changes in living cells while monitoring cell toxicity of the candidate compounds at the same time. Finally, the relatively high temporal resolution of the spectral FRET assay (2–3 seconds per image, compared to 3–4 min per image in the FLIM assay) enables near “real time” monitoring of changes in the PS1/γ-secretase conformation during experimental manipulations.

These data show directly that changes in PS1 conformation can be detected following various manipulations of PS1 itself, Pen2, Aph1, APP, and pharmacological agents known as γ-secretase modulators. Together these results strongly support the hypothesis that γ-secretase catalytic specificity can be allosterically modified via conformational changes in the catalytic PS1 moiety. Therefore, monitoring PS1 conformation using the G-PS1-R probe could be a powerful approach to uncover pathological mechanisms leading to increased Aβ_42/40_ ratio *in vitro* and in mouse brain *in vivo*, as well as a promising tool for therapeutic drug screening of γ-secretase modulating agents.

## Materials and Methods

### Cell Lines and Pharmacological Treatments

Chinese hamster ovary (CHO) cells and Human embryonic kidney (HEK) 293 cells were obtained from ATCC (Manassas, VA). PS1/PS2 double knockout mouse embryonic fibroblasts (PS1/2 dKO MEF) as well as Aph1ABC^−/−^ cells, reconstituted with either Aph1A_L_ or Aph1B component were previously described [14], [34]. CHO, HEK293 and MEF cells were maintained in Opti-MEM (Invitrogen) supplemented with 5% fetal bovine serum. The cells were plated into four-chamber slides or 35 mm glass-bottom dishes, transfected or infected with various constructs, and were used for biochemical (Western blot and ELISA) or microscopy (FLIM, spectral FRET, TIRFM) analyses. The cells were treated for 24 hours with 100 µM fenofibrate or 400 µM ibuprofen to evaluate the effect of Aβ_42_ –modulating compounds on PS1 conformation. Dimethylsulfoxide (DMSO) or ethanol were used as respective vehicle controls.

### Constructs and Lenti-Viral Infections

The GFP-PS1-RFP (G-PS1-R) construct was generated as follows: Human PS1 (NM_000021) was cloned into EGFP-C3 vector using XhoI and HindIII restriction sites to generate N-terminally (NT) labeled GFP-PS1 construct (G-PS1). A NotI restriction site was introduced after the G-PS1 codon 351 within the cytoplasmic loop between transmembrane domains 6 and 7 (TM6-7), resulting in G-PS1_NotI_ construct (based on the cloning strategy by Drs. Haass and Kaether, Munich, Germany). NotI-RFP-NotI tag was produced by polymerase chain reaction and sub-cloned into G-PS1_NotI_ to generate the GFP-PS1-RFP (G-PS1-R) construct. The familial Alzheimer's disease L166P PS1 mutation was introduced into the G-PS1-R construct using a QuickChange site-directed mutagenesis kit (Stratagene, La Jolla, CA) according to the manufacturer's instructions. RFP was also inserted into the loop domain of wtPS1 construct to generate PS1-R plasmid. The integrity of constructs was confirmed by sequencing. To increase the efficiency of G-PS1-R construct delivery to cells, it was packaged into lentivirus (MGH Viral Core Facility, Director Dr. Miguel Sena-Esteves). For the fluorescent lifetime imaging microscopy (FLIM) analysis, G-PS1-R lenti-virus multiplicity of infection, (MOI), was 0.2-1 for CHO, HEK293 and MEF cells. For the Western blotting of G-PS1-R infected CHO or MEF cells we used G-PS1-R lentis at MOI 10. Alternatively, a G-PS1-R encoding plasmid was transfected or co-transfected with other plasmid constructs as indicated in the manuscript using Lipofectamine 2000 (Invitrogen). N-terminally truncated Notch 1 receptor construct (NEC), which does not require ligand binding for its activation and represents an immediate substrate of γ-secretase was a gift from Dr. R. Kopan [35]. Pen2 construct tagged with Flag on its N-terminus was a gift from Dr.Selkoe (BWH, Boston, MA). G-PS1 construct as well as EGFP-C3 empty vector (Clontech, Madison, WI) was used as negative controls for the Forster resonance energy transfer (FRET) based experiments. An RFP-GFP fusion (R-G fusion) plasmid in which RFP is fused to the N-terminus of GFP with a short linker was used as a positive control in the FRET-based assays.

### Western Blot Analysis

For the Western blot analysis CHO or MEF cells plated on 100 mm cell culture dishes were infected with G-PS1-R for 48–72 hrs. Where NEC needed to be co-expressed, the MEF cells were co-transfected with NEC 24 hrs prior to the cell harvesting. The cells were lysed in 1% CHAPSO, and the lysates were resolved on a 4–20% Tris-Glycine gel. The following antibodies were used for detection: rabbit anti-APP C-terminus [36](C8, Selkoe et al., 1988), rabbit anti-GFP (Clontech); mouse anti-PS1 loop (Chemicon), rabbit anti-NICD (Val1744, Cell Signaling).

### Enzyme-Linked ImmunoSorbent Assay (ELISA)

For the detection of Aβ production, we used PS70 cells, CHO cells stably transfected with wild type human APP and PS1 [37]. PS70 cells growing in 35 mm cell culture dishes were treated with drugs or transiently transfected with plasmids encoding either wt Pen-2 or Flag-Pen-2 using Lipofectamine 2000 reagent (Invitrogen). Sixteen hours after the transfection cells culture media was exchanged to 1 ml of Opti-MEM (Invitrogen) supplemented with 2% fetal bovine serum, and the cells were incubated for additional 24 hours. The cell culture media for each transfection condition was subjected to the analysis of human Amyloid β (40) and (42) using ELISA assay kit (WAKO), according to the manufacturer's instructions.

### Fluorescence Lifetime Imaging Microscopy (FLIM)

A FLIM assay was employed to monitor proximity between the PS1 NT and TM6-7 loop as an indicator of PS1 conformation in live cells expressing the G-PS1-R construct. A donor fluorophore lifetime is an indicator of the proximity between the donor and an acceptor fluorophore. If the fluorophores are in close proximity to each other (<10 nm), the GFP donor lifetime decreases due to a non-radiative transfer of part of its emission energy to the RFP acceptor (FRET present). GFP fused to the PS1 NT and RFP located in the PS1 TM6-7 loop serve as FRET donor and acceptor fluorophores, respectively. The GFP-PS1 construct, in which the donor lifetime is measured in the absence of an acceptor (FRET absent), was used as a negative control. GFP-RFP fusion construct was used as FRET positive control.

Live cells were imaged 2–4 days post-transfection/infection on a Zeiss LSM510 microscope equipped with 37°C heating chamber containing 5% CO_2_. A Chameleon Ti∶Sapphire laser was used to excite the donor fluorophore (two-photon excitation wave length: 840 nm for GFP donor and 800 nm for Alexa488 donor), a high-speed Hamamatsu (MCP R3809; Hamamatsu, Hamamatsu City, Japan) detector with very high temporal resolution optimized for fluorescence lifetime imaging, and Becker&Hickl TCSPC hardware/software (Becker&Hickl, Berlin, Germany) were used to acquire donor lifetime information on a pixel-by-pixel basis. Data analysis was performed using SPC Image (Becker&Hickl, Berlin, Germany), in which donor fluorophore lifetimes are determined by fitting the data to one (negative control) or two (experimental conditions) exponential decay curves. A 128×128 pixel matrix was created to display donor lifetimes on a pixel-by-pixel basis in pseudocolored images, in which red pixels represent molecules in close proximity (FRET present), whereas blue pixels represent molecules that are greater than 10 nm apart from each other (FRET absent).

Alternatively, PS70 cells or CHO cells transfected with untagged wild type PS1 were fixed in 4% paraformaldehyde and incubated with antibodies against PS1 NT and C-terminus (CT) or TM 6–7 loop domain as previously described [21]. PS1 NT was labeled with Alexa488 (donor) and PS1 CT/PS1 loop with Cy3 (acceptor) to be subjected to the FLIM analysis as described above. The following antibodies were used for immunostaining; goat anti-PS1 NT (Sigma), rabbit anti-PS1 CT (Sigma), mouse anti-PS1 loop (Chemicon), donkey anti-goat Alexa 488 (Molecular probe), donkey anti-rabbit Cy3 (Molecular probe), and donkey anti-mouse Cy3 (Molecular probe).

### Total Internal Reflection Fluorescence Microscopy (TIRFM)

To test whether G-PS1-R protein traffics through the secretory pathway and reaches the plasma membrane, we performed a TIRFM assay. TIRFM detects fluorescent proteins within or in close proximity (≤100 nm) to the plasma membrane of living cells. The high resolution is achieved by laser beam being totally reflected on a cell-substrate interface. Thus, only a very thin layer of the cell immediately adjacent to the interface between two media is illuminated by the evanescent electromagnetic field. Cells were transfected with the G-PS1-R constructs and then re-plated a day before the imaging on a glass cover slip at 30% confluency using media that did not contain phenol red. The cells were placed in a CO_2_ and temperature controlled chamber of Carl Zeiss system for TIRF imaging. The excitation of EGFP was used to study presence of the GFP-PS1-RFP expression product within the proximity of the plasma membrane. Modified Carl Zeiss system was used to record the images as previously described [38].

### Spectral FRET

HEK293 cells in 35 mm glass-bottom dishes were transfected with PS1 constructs fused with living color tags (G-PS1, G-PS1-R or L166P G-PS1-R). RFP-GFP fusion construct was used as a FRET positive control. Cells were imaged 24–48 hours after the transfection. Spectral imaging was performed on the Zeiss LSM510 microscope equipped with 37°C heating chamber containing 5% CO_2_. The Argon laser at 488 nm was used to excite GFP, and emitted fluorescence was detected by 7 channels of the Metadetector with 21.4 nm spectral bandwidth for each channel that was set to detect fluorescence in the 502–651 nm wavelength range. The images were processed and quantified using LSM image browser as follows: average pixel intensity of the fluorescence in the entire cell was determined for each spectral window. G-PS1 transfected cells (negative control) showed maximal fluorescence emission at 513 nm. When FRET occurs, the energy is transferred from GFP (donor) to RFP (acceptor), thus the GFP emission peak weakens, and the fluorescence intensity of RFP increases. The FRET positive control probe (R-G fusion) showed robust increase in the fluorescence intensity at 598 nm, compared to that of the FRET negative control (G-PS1). Thus, we used the ratio of 598 nm (RFP) to 513 nm (GFP) as a readout of the FRET efficiency, which reflects relative proximity between the GFP and RFP fluorophores. The ratio value for the whole cell was calculated as a ratio of average pixel fluorescence intensity at the 598 nm spectral window to that at the 513 nm, after subtraction of the background fluorescence. We used 10x and 25x objectives for the average-per-cell analysis, and 63X objective for studying sub-cellular distribution of PS1. The detector gain and an amplifier offset were adjusted to make sure that the intensities of cells in the image were not saturated and the setting maintained the same for all experimental manipulations. Ratiometric image was produced by dividing the intensity of the image from the 598 nm spectral window by that from the 513 nm window after background subtraction, using Image J. After adjustment of brightness/contrast, a Look-Up Table was applied by mapping image values to color-scale (16 colors) resulting in pseudo-color image.

### Statistical Analysis

StatView for Windows, Version 5.0.1 (SAS Institute, Inc) was employed to perform statistical analysis using Fisher's PSLD analysis of variance (ANOVA). Samples were considered significantly different at p<0.05. For the comparison of 598/513 ratio in spectral FRET assay, a t-test was used for the analysis.

## Supporting Information

Figure S1 Expression of G-PS1-R probe in HEK 293 cells. (A–C) G-PS1-R probe was transfected in HEK293 cells and observed by conforcal microscopy. GFP (A) as well as RFP signal (B) was observed mainly in perinuclear area in reticular pattern, reminiscent of ER distribution. (C) Merged image.
(1.56 MB TIF)

Figure S2 Conformational diversity of the G-PS1-R molecules in different subcellular compartments. (A) - Confocal image of the GFP fluorescence in HEK293 cell infected with G-PS1-R-lenti viral construct. Several ROIs (circles) were randomly chosen and 598/513 ratio was calculated for each ROI. The 598/513 ratio in the each ROI was shown next to the circle. Higher ratio represents closer GFP-PS1 NT to RFP-PS1-loop proximity. (B)- Spectral FRET pseudo-colored image is produced by dividing fluorescence intensity of the image in the 598 spectral window by that in the 513 window on a pixel-by-pixel basis after background subtraction. The colorimetric scale bar shows 598/513 ratios from 0 (black, GFP and RFP far apart) to 0.5 (white, GFP-RFP close together). (C) - FLIM pseudo-color image of the GFP lifetime distribution in HEK293 cells transfected with G-PS1-R. The colorimetric scale bar shows lifetime in picoseconds. Note red pixels (shorter lifetime) are predominantly located at/near the cell surface.
(1.56 MB TIF)
