# Supplementary figures and images for: Allosteric Modulation of PS1/γ-Secretase Conformation Correlates with Amyloid β42/40 Ratio
Source: PLoS One. 2009 Nov 18;4(11):e7893. doi: 10.1371/journal.pone.0007893 (PMC2773935; doi:10.1371/journal.pone.0007893)

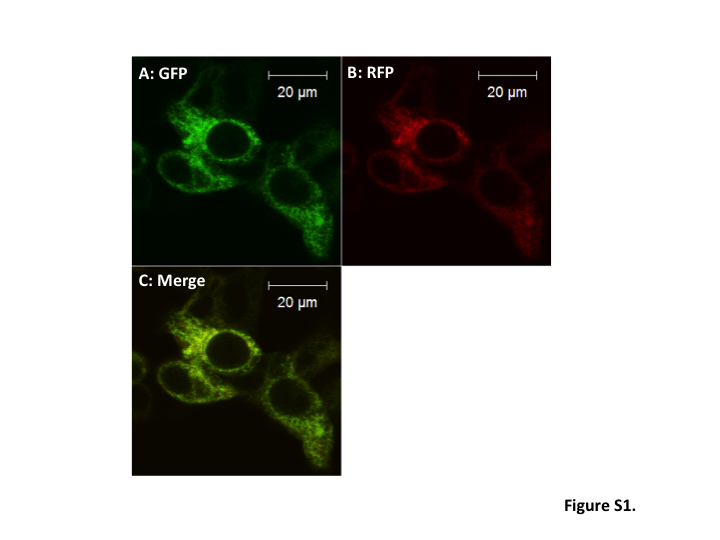

Supplement: Figure S1 — Expression of G-PS1-R probe in HEK 293 cells. (A–C) G-PS1-R probe was transfected in HEK293 cells and observed by conforcal microscopy. GFP (A) as well as RFP signal (B) was observed mainly in perinuclear area in reticular pattern, reminiscent of ER distribution. (C) Merged image. (1.56 MB TIF) [file pone.0007893.s001.tif]

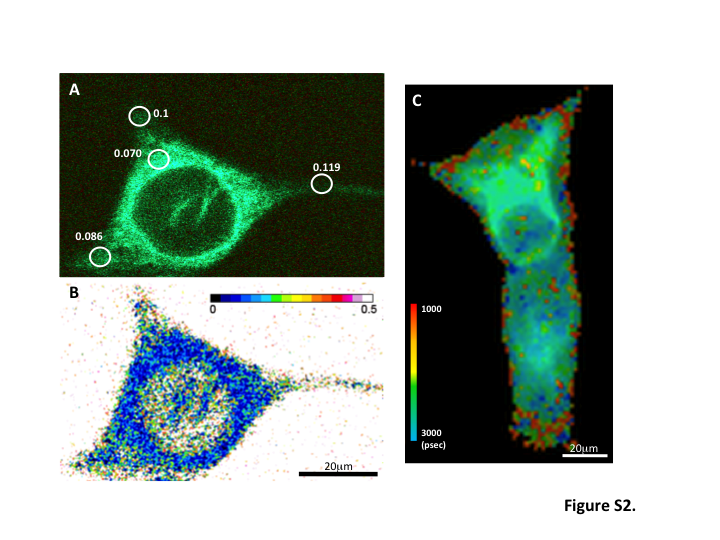

Supplement: Figure S2 — Conformational diversity of the G-PS1-R molecules in different subcellular compartments. (A) - Confocal image of the GFP fluorescence in HEK293 cell infected with G-PS1-R-lenti viral construct. Several ROIs (circles) were randomly chosen and 598/513 ratio was calculated for each ROI. The 598/513 ratio in the each ROI was shown next to the circle. Higher ratio represents closer GFP-PS1 NT to RFP-PS1-loop proximity. (B)- Spectral FRET pseudo-colored image is produced by dividing fluorescence intensity of the image in the 598 spectral window by that in the 513 window on a pixel-by-pixel basis after background subtraction. The colorimetric scale bar shows 598/513 ratios from 0 (black, GFP and RFP far apart) to 0.5 (white, GFP-RFP close together). (C) - FLIM pseudo-color image of the GFP lifetime distribution in HEK293 cells transfected with G-PS1-R. The colorimetric scale bar shows lifetime in picoseconds. Note red pixels (shorter lifetime) are predominantly located at/near the cell surface. (1.56 MB TIF) [file pone.0007893.s002.tif]
